# Time trends in depression prevalence and health-related correlates: results from population-based surveys in Germany 1997–1999 vs. 2009–2012

**DOI:** 10.1186/s12888-018-1973-7

**Published:** 2018-12-20

**Authors:** Julia Bretschneider, Silke Janitza, Frank Jacobi, Julia Thom, Ulfert Hapke, Tobias Kurth, Ulrike E. Maske

**Affiliations:** 1 Unit 26 Mental Health, Department of Epidemiology and Health Monitoring, Robert Koch-Institute, Berlin, Germany; 2 Psychologische Hochschule Berlin, Berlin, Germany; 3 Institute of Public Health, Charité – Universitätsmedizin Berlin, Berlin, Germany

**Keywords:** Major depressive disorder, Depression, Prevalence, Time trends, Health-related quality of life, Disease burden, General population, Germany, Epidemiology, Cross-sectional studies

## Abstract

**Background:**

Although an “epidemic” of depression is frequently claimed, empirical evidence is inconsistent, depending on country, study design and depression assessment. Little is known about changes in depression over time in Germany, although health insurance companies report frequency increases. Here we examined time trends in depression prevalence, severity and health-related correlates in the general population.

**Methods:**

Data were obtained from the mental health module of the “German Health Interview and Examination Survey for Adults” (2009–2012, *n* = 3265) and the mental health supplement of the “German National Health Interview and Examination Survey 1998” (1997–1999, *n* = 4176), excluding respondents older than 65. 12-month major depressive disorder (MDD), severity and symptoms were assessed based on the WHO Composite International Diagnostic Interview. Health-related quality of life (SF-36), self-reported sick days or days with limitations in normal daily life activities were examined, too. Calculations were carried out population-weighted. Additional age-standardized analyses were conducted to account for demographic changes.

**Results:**

Overall, MDD 12-month prevalence remained stable at 7.4%. Women showed a shifted age distribution with increased prevalence at younger ages, and increasing MDD severity. Time trends in health-related correlates occurred both in participants with and without MDD. Mental health disability increased over time, particularly among men with MDD, reflected by the mental component score of the SF-36 and days with activity limitation due to mental health problems. Demographic changes had a marginal impact on the time trends.

**Conclusions:**

In contrast to the ongoing international debate regarding increased depression rates in western countries, we found no increase in overall MDD prevalence in Germany over a long period. In conclusion, increased depression frequencies in national health insurance data and growing health care costs associated with depression are not attributable to overall prevalence changes at a population level. However, shifted age distribution and increased severity among women may reflect a rising depression risk within this specific subgroup, and changes in health-related correlates indicate a growing mental health care need for depression, particularly among men.

**Electronic supplementary material:**

The online version of this article (10.1186/s12888-018-1973-7) contains supplementary material, which is available to authorized users.

## Background

An “epidemic” of depression as a disease of modernity has been frequently claimed [1–3]. Given the high prevalence and enormous personal and economic disease burden, this discourse highlights a global core health challenge of the 21st century [4–6].

According to the World Health Organization (WHO), over 300 million people were estimated to be affected by depression globally in 2015, an increase of 18.4% since 2005 [7]. However, changes were mainly attributable to the overall growth of the global population and its changing age structure [7, 8]. Likewise, a meta-analysis of 116 epidemiological studies revealed no change in the prevalence of major depressive disorder (MDD) between 1990 and 2010 using age-adjusted estimates, challenging the notion of an epidemic [3]. However, the empirical evidence is inconsistent, depending on country, study design, disorder definition and assessment of depression, and several previous studies also indicate a slight increase of depressive symptoms over time [9–12]. The evaluation of time trends based on direct comparisons of population-based data is difficult, and different depression measures lead to varying results [1]. Even if assessed with a fully-structured clinical diagnostic interview based on diagnostic criteria of the Diagnostic and Statistical Manual of Mental Disorders (DSM), the “gold standard” for estimating depression prevalence [13, 14], prevalence estimates are affected by minor changes in the diagnostic criteria and revisions of clinical interviews or diagnostic algorithms used in mental health survey replications [15]. Thus, there is little agreement about whether depression prevalence is increasing over time or not. However, there is no evidence for a reduction in prevalence [16, 17].

In Germany, little is currently known about changes in depression prevalence over time. However, health insurance companies have reported an increasing depression frequency [18], and health care costs attributable to depression have increased considerably [19]. Besides the possibility of increasing depression prevalence in the general population, time trends in health insurance data could also reflect a rising need for mental health care (e.g., due to increasing functional disability). Therefore, the current study sought to examine changes over a period of 13 years for MDD prevalence, severity, symptoms, and health-related correlates for the general population in Germany.

## Methods

### Study design and population

Mental disorders, including MDD, were assessed in the mental health module of the first wave of the “German Health Interview and Examination Survey for Adults” (DEGS1-MH, data collection 2009–2012, age range 18–79 years) and the mental health supplement of its predecessor, the “German National Health Interview and Examination Survey 1998” (GHS-MHS, data collection 1997–1999, age range 18–65 years). Both surveys were part of the German health monitoring system at the Robert Koch-Institute. They provide representative nationwide data about the health of the non-institutionalized adult population in Germany. Design and methods are described in detail elsewhere [20–24]. In brief, the core surveys GHS and DEGS1 were conducted using two-stage clustered random sampling (step 1: random selection of study locations from all municipal communities; step 2: random selection of participants from population-registries in each selected study location). The net samples of GHS (*n* = 7124, response rate: 61%) and DEGS1 (*n* = 7115, including 3959 participants who participated in both surveys, response rates: 64% among former GHS participants and 42% for newly sampled individuals) enable representative cross-sectional and trend analyses [25]. The mental health assessment was completed by *n* = 4181 participants in GHS (conditional response rate: 87.6%, see [23]) and *n* = 4483 participants in DEGS1 (conditional response rate: 88.2%, see [24]). Excluding participants with missing data regarding 12-month MDD from both surveys and participants older than 65 from DEGS1-MH, the final study sample was *n* = 4176 for GHS-MHS and *n* = 3265 for DEGS1-MH.

### Assessment of depression

Major depressive disorder (MDD) during the last 12 months was assessed by trained interviewers based on the WHO Composite International Diagnostic Interview (CIDI). The CIDI is a standardized fully-structured computer-assisted clinical face-to-face interview. It is widely used internationally in the assessment of mental disorders according to the diagnostic criteria of the DSM [13, 26, 27]. A modified German version of the CIDI, the Munich Composite International Diagnostic Interview, was used in the GHS-MHS (DIA-X/M-CIDI; [28]) and modified for DEGS1-MH (DEGS-CIDI; [24]) to assess mental disorders, according to the diagnostic criteria of the DSM-IV and DSM-IV-TR, respectively [29]. MDD requires the persistence of at least five of nine depression symptoms on nearly every day for 2 weeks or longer, of which at least one is depressed mood or decreased interest/pleasure (DSM criterion A). Furthermore, clinically significant distress and impairment associated with these symptoms are necessary (DSM criterion C). MDD exclusion criteria include lifetime manic/hypomanic episodes and depressive symptoms solely attributable to the direct physiological effects of a substance, a general medical condition or attributable to grief. The questions related to depression assessment were similar in the versions of the CIDI in both the GHS-MHS and DEGS1-MH. However, skip rules, and diagnostic algorithms for deduction of 12-month MDD diagnosis differed slightly. Therefore, trend analyses were based on a specific unified diagnostic algorithm. In brief, the algorithm for 12-month MDD was limited to information about the last 12 months without considering lifetime information on symptoms and disorders, and the operationalization of exclusion criteria was harmonized between DEGS1-MH and GHS-MHS. This modified algorithm enables the estimation of prevalence changes over time but also leads to slightly different estimates compared with previously published MDD prevalence data for Germany [30–32]. For participants with 12-month MDD, depression severity was categorized based on the number of depression symptoms into “mild” (5 symptoms), “moderate” (6–7 symptoms) and “severe” (8–9 symptoms) (see [33]).

### Other measures

In the core surveys (GHS and DEGS1), socio-demographic variables were assessed, including sex, marital status (married and living with partner, married and not living with partner, single, divorced, widowed), socio-economic status (SES; classified in low, middle and high based on information on education, occupational, status and net household income, see [34]) and community size [categorized in rural (< 5000 inhabitants), small town (5000 to < 20,000), mid-sized town (20,000 to < 100,000) and large town, see [25]. Age was assessed in years at the time of mental health assessment (GHS-MHS and DEGS1-MH) and categorized into age groups (18–34, 35–49 and 50–65).

Self-rated health and health-related quality of life (past 4 weeks) were assessed with a self-administered questionnaire using the German version of the Short Form 36 (SF-36) version 1 in the GHS [35, 36] and version 2 in the DEGS1 [37, 38]. The first question in the SF-36 (identical in both versions) measuring self-rated health was dichotomized into poor/fair vs. good/very good/excellent (see [39]). The SF-36 distinguishes eight domains for health-related quality of life: physical functioning, role physical, bodily pain, general health, vitality, social functioning, role emotional and mental health. A physical component score (PCS) and a mental component score (MCS) are constructed as total scales. “Norm-based scoring” enabled comparability between SF-36 versions (see [37]). Thus, SF-36 scales of both versions were standardized to the 1998 American normative random sample then transformed to an average value of 50 and a standard deviation of 10. Higher values indicate better health-related quality of life.

The number of days with limitations in normal daily life activities due to mental or physical health problems (past 4 weeks) were assessed in the same way in both survey mental health modules [23, 24]: participants were asked on how many days during the past 4 weeks they were totally limited in daily life activities due to mental and physical health problems. In this study, answers were dichotomized into “no” vs. “any” days with limitation. The number of sick days (past 12 months) was assessed in an identical way in both survey mental health modules using a self-administered questionnaire [21, 40] and dichotomized into “no” vs. “any” sick days for this study.

### Statistical analysis

All statistical analyses were performed using survey-specific weighting factors adjusting the study samples to the demographic-geographic distribution of the population in Germany, as on 12/31/1997 (for GHS-MHS) and 12/31/2010 (for DEGS-MH). Adjustment took sex, age, educational status, federal state, and nationality into account, as well as the probability of re-participation in the mental health module subsequent to the core survey [23–25]. Statistical analyses were performed using Stata 14.1 and survey design procedures accounting for clustering and weighting. Statistical significance was based on a two-sided significance level of 0.05. Analyses were not adjusted for multiple testing. Prevalence, frequencies, means and 95% confidence intervals (95%-CI) for all measures were reported for each sex for participants aged 18–65 years in the GHS-MHS and DEGS1-MH. In addition, estimates from age-standardized analyses were reported where appropriate. Note that the provided age-standardized values cannot be interpreted as valid cross-sectional population estimates themselves, but reflect changes over time that are unaffected by demographic changes in the underlying population. To calculate age-standardized prevalence estimates, participants from the GHS-MHS were weighted to the demographic-geographic population structure underlying the DEGS1-MH (as on 12/31/2010).

Distribution of depression severity and prevalence of depression symptoms were reported for cases with 12-month MDD. The Rao-Scott chi-square test was used to test time trends. Age-adjusted results are described for depression severity based on multinomial logistic regression model with depression severity as dependent variable including time point (GHS-MHS vs. DEGS1-MH) and age as independent variables (reference: 1997–1999). Health-related correlates are reported for participants with and without 12-month MDD to enable evaluation of time trends for MDD cases compared with the remaining population. The number of sick days was examined if participants reported any sick days. Effect estimates for time trends were calculated based on linear, logistic and negative binomial regression models, including MDD, time point (GHS-MHS vs. DEGS1-MH) and the interaction between MDD and time point as independent variables (reference: 1997–1999). Results of additional age-adjusted regression models are supplementary and described only if divergent from unadjusted results.

## Results

Demographic features of GHS-MHS and DEGS1-MH are shown in Table 1. Overall, the sample characteristics showed little change in the underlying population between 1997–1999 and 2009–2012 regarding the included variables, except for age distribution (shift towards older age) and marital status (being married and living with a partner became less frequent, while the proportion of singles increased).

**Table 1:** Sample characteristics of GHS-MHS (1997–1999)^1^ and DEGS1-MH (2009–2012)^2^

	Men		Women	
	GHS-MHS (*n* = 1911)	DEGS1-MH (*n* = 1522)	GHS-MHS (*n* = 2265)	DEGS1-MH (*n* = 1743)
Age group (years), % (95%-CI)				
18–34	36.4 (33.5–39.5)	30.0 (26.9–33.2)	35.4 (33.0–37.9)	31.3 (28.8–34.0)
35–49	34.0 (31.4–36.7)	36.0 (33.1–39.0)	33.3 (31.0–35.8)	36.1 (33.4–39.0)
50–65	29.6 (27.0–32.3)	34.0 (31.3–36.9)	31.3 (29.3–33.4)	32.5 (30.0–35.2)
Socioeconomic status^3^, % (95%-CI)				
Low	17.0 (14.7–19.6)	18.2 (15.4–21.3)	17.6 (15.4–20.1)	16.9 (14.5–19.6)
Medium	61.4 (58.8–63.9)	58.3 (54.9–61.6)	62.9 (60.4–65.4)	63.1 (59.5–66.6)
High	21.7 (19.1–24.5)	23.5 (20.8–26.5)	19.4 (17.2–21.9)	20.0 (17.6–22.6)
Community size^4^, % (95%-CI)				
Rural (< 5000 inhabitants)	20.8 (14.0–29.8)	15.9 (10.6–23.2)	18.9 (12.6–27.4)	14.5 (9.4–21.5)
Small town (5000 to < 20,000)	21.9 (14.7–31.5)	23.8 (17.4–31.7)	19.9 (13.3–28.5)	24.0 (17.6–31.8)
Mid-sized town (20,000 to < 100,000)	26.7 (19.1–36.0)	29.2 (22.1–37.6)	28.7 (20.7–38.3)	29.8 (22.7–37.9)
Large town (≥ 100,000)	30.6 (22.5–40.1)	31.0 (23.8–39.3)	32.6 (24.1–42.4)	31.8 (24.5–40.1)
Marital status, % (95%-CI)				
Married and living with partner	63.3 (60.2–66.2)	57.9 (54.4–61.2)	65.3 (62.2–68.3)	59.2 (55.8–62.4)
Married and not living with partner	1.9 (1.2–3.0)	1.7 (1.0–3.0)	2.9 (2.1–3.9)	2.5 (1.6–3.7)
Single (never been married)	28.9 (26.3–31.7)	34.3 (31.2–37.6)	20.7 (18.5–23.0)	28.1 (25.3–31.0)
Divorced	4.9 (3.9–6.3)	5.2 (3.9–6.9)	6.7 (5.4–8.3)	6.9 (5.6–8.6)
Widowed	1.0 (0.6–1.6)	0.9 (0.4–1.9)	4.4 (3.4–5.7)	3.4 (2.5–4.7)

^1^German National Health Interview and Examination Survey 1998, mental health supplement (GHS-MHS, 1997–1999): weighted for population structure as of 12/31/1997; age range: 18–65; *n* = 4176 with full mood disorders section within the Composite International Diagnostic Interview (CIDI)

^2^German Health Interview and Examination Survey for Adults, mental health module (DEGS1-MH, 2009–2012): weighted for population structure as of 12/31/2010; age range: 18–65; *n* = 3265 with full mood disorders section within the Composite International Diagnostic Interview (CIDI)

^3^Based on information regarding education, occupational status and net household income

^4^GHS-MHS: community size as of 12/31/1996; DEGS1-MH: community size as of 12/31/2006

### 12-month prevalence of MDD

**Fig. 1:**
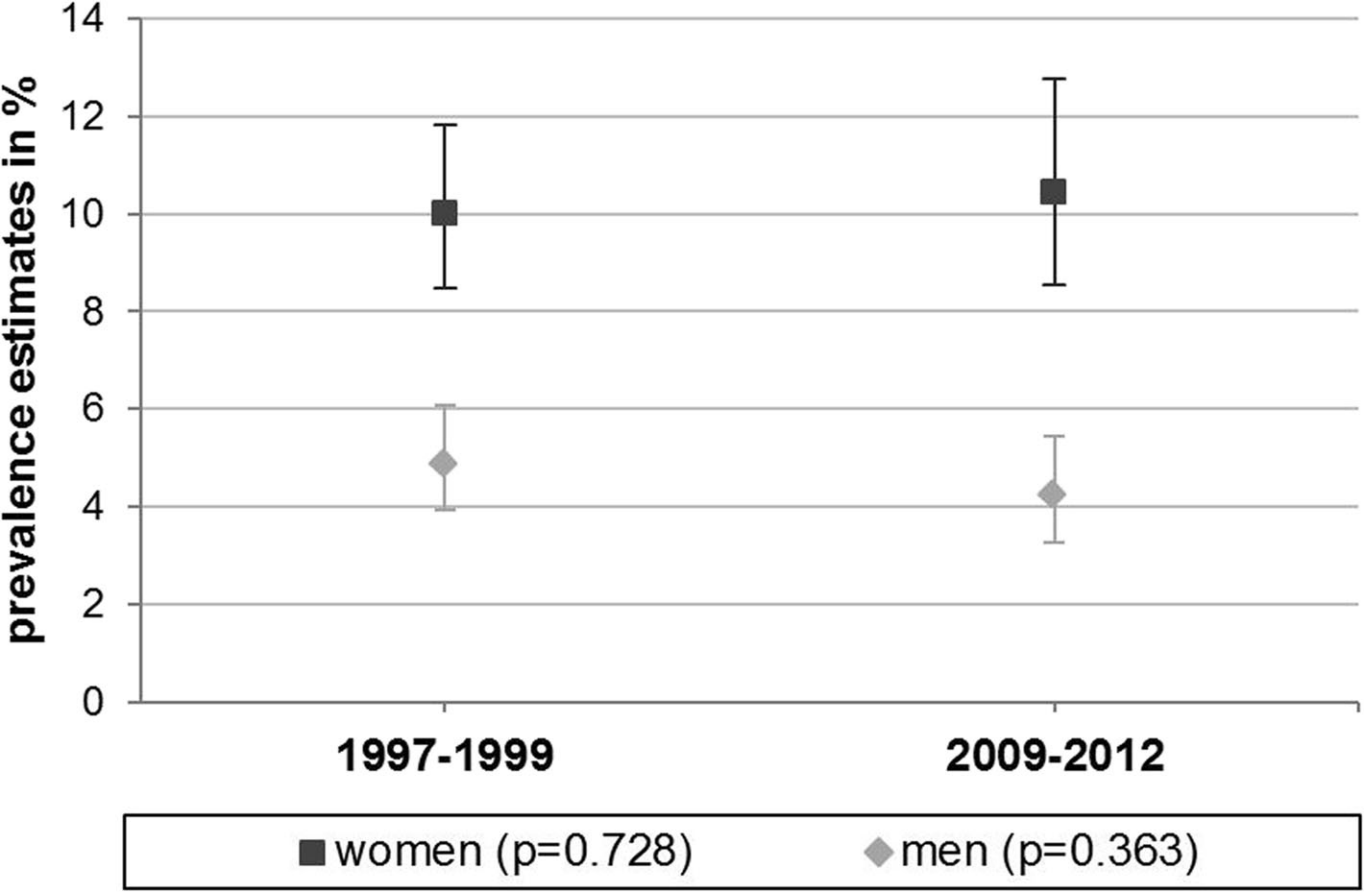
12-month prevalence of MDD 1997–1999 (GHS-MHS: German National Health Interview and Examination Survey 1998, mental health supplement, weighted for population structure as of 12/31/1997) vs. 2009–2012 (DEGS1-MH: German Health Interview and Examination Survey for Adults, mental health module, weighted for population structure as of 12/31/2010). Age range: 18–65; *p*-values: Rao-Scott chi-square test for testing differences between 1997–1999 and 2009–2012

Overall 12-month MDD prevalence was stable over time (GHS-MHS: 7.4%, 95%-CI: 6.5–8.5 vs. DEGS1-MH: 7.4%, 95%-CI: 6.1–8.8; *p* = 0.93 when testing for differences), as well as age-standardized prevalence (GHS-MHS: 7.4%, 95%-CI: 6.4–8.6 vs. DEGS1-MH: 7.4%, 95%-CI: 6.1–8.8; *p* = 0.96). Although prevalence estimates slightly increased in women and decreased in men, these changes were not statistically significant (see Fig. 1). This was also the case with age-standardization (women: GHS-MHS: 9.6%, 95%-CI: 8.0–11.5 vs. DEGS1-MH: 10.5%, 95%-CI: 8.6–12.8; *p* = 0.53; men: GHS-MHS: 5.2%, 95%-CI: 4.1–6.5 vs. DEGS1-MH: 4.2%, 95%-CI: 3.3–5.4; *p* = 0.22). There were no significant sex differences regarding prevalence trends (logistic model with MDD as dependent variable, *p* = 0.30 for testing the interaction between sex and time point, resp. 0.13 with age-standardization). Prevalence among women was significantly higher than among men at both time points.

There were significant age-specific time trends for women (see Table 2): 12-month prevalence of MDD increased in women aged 18 to 34 years (*p* = 0.005) from 8.8% (95%-CI: 6.6–11.6) to 15.6% (95%-CI: 11.3–21.0) and decreased in the oldest age group (*p* = 0.002) from 9.8% (95%-CI: 7.3–13.0) to 5.0% (95%-CI: 3.5–7.1). In contrast, for women aged 35 to 49 years the prevalence remained constant, at approximately 11%. For men, prevalence estimates did not significantly differ within age groups. There were also no significant changes over time depending on socioeconomic status for men and women. Further analyses revealed similar significant differences and comparable prevalence estimates within age groups, and, with regard to socioeconomic status, when controlling for sociodemographic variables (logistic models adjusting for socioeconomic status, or age, as well as community size and marital status; results not shown).

**Table 2:** 12-month prevalence of MDD 1997–1999^1^ vs. 2009–2012^2^ by age group and socioeconomic status

	Men			Women		
	1997–1999 (*n* = 1911)	2009–2012 (*n* = 1522)	*p*-value^3^	1997–1999 (*n* = 2265)	2009–2012 (*n* = 1743)	*p*-value^3^
Total, % (95%-CI)	4.9 (3.9–6.1)	4.2 (3.3–5.4)	0.363	10.0 (8.5–11.8)	10.5 (8.6–12.8)	0.728
Age group (years), % (95%-CI)						
18–34	4.4 (3.0–6.5)	4.0 (2.4–6.7)	0.761	**8.8 (6.6–11.6)**	**15.6 (11.3–21.0)**	**0.005**
35–49	5.4 (3.9–7.5)	4.0 (2.4–6.7)	0.333	11.5 (9.2–14.3)	11.0 (8.1–14.7)	0.787
50–65	4.9 (3.4–6.9)	4.6 (3.1–6.8)	0.812	**9.8 (7.3–13.0)**	**5.0 (3.5–7.1)**	**0.002**
SES, % (95%-CI)						
Low	5.6 (2.9–10.5)	5.1 (2.7–9.5)	0.864	11.7 (8.4–16.0)	17.0 (11.1–25.2)	0.128
Middle	5.3 (4.1–6.9)	4.4 (3.1–6.2)	0.331	10.1 (8.2–12.3)	9.3 (7.1–12.1)	0.610
High	3.3 (2.2–5.0)	3.1 (1.7–5.7)	0.874	8.6 (6.4–11.5)	8.6 (5.6–12.7)	0.981

^1^German National Health Interview and Examination Survey 1998, mental health supplement (GHS-MHS, 1997–1999): weighted for population structure as of 12/31/1997; age range: 18–65

^2^German Health Interview and Examination Survey for Adults, mental health module (DEGS1-MH, 2009–2012): weighted for population structure as of 12/31/2010; age range: 18–65

^3^*p*-value based on Rao-Scott chi-square test. Bold type indicates significant differences between 1997–1999 and 2009–2012 (local significance level α = 0.05)

### Distribution of depression severity and depression symptoms in cases with 12-month MDD

Overall, for men and women with 12-month MDD, moderate severity was most frequent (> 40% at both time points). There was a slight decrease in mild depression over time, while severe depression increased (see Table 3). However, these changes were statistically significant only for women with mild depression, decreasing from 24.1% to 13.8% (*p* = 0.045). Comparable results were obtained when controlling for age in a multinomial logistic model (results not shown). Furthermore, differences observed were not attributable to changes only within specific age groups (results not shown).

**Table 3:** Depression severity in cases with 12-month MDD 1997–1999^1^ vs. 2009–2012^2^

	Men			Women		
	1997–1999 (*n* = 110)	2009–2012 (*n* = 71)	*p*-value^3^	1997–1999 (*n* = 238)	2009–2012 (*n* = 159)	*p*-value^3^
Depression severity, % (95%-CI)						
Mild (5/9 symptoms)	31.7 (22.3–42.9)	27.1 (16.2–41.7)	0.574	**24.1 (18.3–30.9)**	**13.8 (8.0–22.7)**	**0.045**
Moderate (6 + 7/9 symptoms)	45.0 (35.2–55.1)	43.0 (31.0–55.9)	0.803	41.5 (34.2–49.2)	47.2 (37.0–57.7)	0.329
Severe (8 + 9/9 symptoms)	23.3 (15.8–33.1)	29.9 (19.0–43.7)	0.373	34.5 (27.2–42.6)	39.0 (28.2–51.1)	0.466

^1^German National Health Interview and Examination Survey 1998, mental health supplement (GHS-MHS, 1997–1999): weighted for population structure as of 12/31/1997; age range: 18–65

^2^German Health Interview and Examination Survey for Adults, mental health module (DEGS1-MH, 2009–2012): weighted for population structure as of 12/31/2010; age range: 18–65

^3^*p*-value based on Rao-Scott chi-square test. Bold type indicates significant differences between 1997–1999 and 2009–2012 (local significance level α = 0.05)

Prevalence of the nine depression symptoms in cases with 12-month MDD was heterogeneous (see Additional file 1). Over time, there was a significant increase in the frequency of reporting a feeling of guilt/worthlessness in men (*p* = 0.035) and women (*p* = 0.019). In addition, women reported significant weight/appetite change more frequently in 2009–2012 (*p* < 0.001) and diminished interest/pleasure less frequently (*p* = 0.018).

### Health-related correlates

Overall, men and women with 12-month MDD exhibited worse outcomes for all health-related correlates than participants without MDD at both time points (see Table 4). Participants with 12-month MDD reported fair/poor self-rated health significantly more often, had significantly lower health-related quality of life in all SF-36 sub-scales, and reported days with activity limitation due to mental health problems and sick days more frequently than participants without MDD.

**Table 4:** Health-related correlates in participants with and without 12-month MDD in 1997–1999^1^ and 2009–2012^2^

	Men				Women			
	With MDD		Without MDD		With MDD		Without MDD	
	1997–1999 (*n* = 110)	2009–2012 (*n* = 71)	1997–1999 (*n* = 1801)	2009–2012 (*n* = 1451)	1997–1999 (*n* = 238)	2009–2012 (*n* = 159)	1997–1999 (*n* = 2027)	2009–2012 (*n* = 1584)
Fair/poor self-rated health, % (95%-CI)	36.8 (26.7–48.3)	37.1 (24.6–51.6)	**13.9 (12.0–16.1)**	**10.1 (8.3–12.3)**	**40.4 (32.7–48.5)**	**21.3 (13.9–31.2)**	**14.6 (12.7–16.6)**	**10.9 (9.2–13.0)**
Health-related quality of life (4 weeks)^3^, mean (95%-CI)								
Physical functioning	50.3 (48.4–52.1)	48.8 (46.0–51.6)	**52.9 (52.5–53.3)**	**53.6 (53.2–54.0)**	47.8 (46.3–49.3)	49.3 (46.9–51.7)	**51.6 (51.1–52.0)**	**52.5 (52.1–52.9)**
Physical role functioning	47.4 (44.8–50.0)	43.1 (39.6–46.6)	52.8 (52.3–53.2)	52.2 (51.7–52.7)	46.5 (44.7–48.2)	46.6 (44.4–48.9)	**51.6 (51.2–52.1)**	**50.7 (50.2–51.2)**
Bodily pain	45.1 (42.6–47.5)	43.4 (40.0–46.7)	**51.1 (50.4–51.7)**	**54.1 (53.4–54.8)**	**41.8 (39.8–43.8)**	**47.9 (45.3–50.6)**	**48.0 (47.4–48.6)**	**51.3 (50.6–52.0)**
General health	42.8 (41.0–44.6)	42.2 (39.3–45.1)	**49.0 (48.5–49.4)**	**50.6 (50.0–51.2)**	43.2 (41.4–44.9)	45.4 (43.3–47.5)	**49.1 (48.6–49.7)**	**50.5 (50.1–51.0)**
Vitality	45.3 (43.2–47.4)	43.1 (39.8–46.3)	52.9 (52.4–53.3)	53.1 (52.6–53.6)	43.0 (41.7–44.3)	43.7 (41.2–46.2)	50.8 (50.3–51.3)	51.2 (50.7–51.8)
Social role functioning	**44.0 (41.7–46.3)**	**39.6 (36.0–43.2)**	52.6 (52.2–53.0)	52.4 (51.9–52.9)	41.4 (39.4–43.4)	41.6 (39.2–44.1)	51.1 (50.6–51.6)	50.9 (50.4–51.5)
Emotional role functioning	**44.6 (41.5–47.7)**	**36.3 (32.4–40.1)**	**52.9 (52.5–53.2)**	**51.6 (51.1–52.1)**	43.1 (40.9–45.3)	40.4 (37.3–43.5)	**52.0 (51.6–52.5)**	**49.4 (48.7–50.0)**
Mental health	39.0 (36.9–41.1)	37.0 (34.1–39.8)	50.2 (49.8–50.7)	50.8 (50.2–51.3)	36.8 (35.0–38.6)	38.7 (36.7–40.8)	**47.6 (47.1–48.2)**	**48.6 (48.0–49.2)**
*Physical component score*	49.2 (47.1–51.2)	48.4 (45.3–51.5)	**51.8 (51.3–52.3)**	**53.4 (52.8–53.9)**	**47.6 (45.9–49.4)**	**51.4 (49.2–53.6)**	**50.6 (50.1–51.1)**	**52.3 (51.9–52.8)**
*Mental component score*	**40.5 (38.0–42.9)**	**35.3 (32.0–38.5)**	**51.6 (51.2–52.0)**	**50.9 (50.4–51.5)**	38.5 (36.5–40.4)	37.2 (34.5–40.0)	**49.8 (49.3–50.4)**	**48.9 (48.3–49.5)**
Any days with activity limitation (4 weeks), % (95%-CI)								
due to mental health problems	**9.1 (4.9–16.5)**	**25.9 (14.8–41.3)**	**0.4 (0.2–0.7)**	**1.7 (1.0–3.0)**	**5.9 (3.1–10.8)**	**29.0 (19.3–41.0)**	**1.6 (1.0–2.6)**	**3.7 (2.6–5.2)**
due to physical health problems	21.0 (13.4–31.2)	26.7 (16.3–40.5)	14.0 (11.8–16.4)	12.7 (10.6–15.2)	20.0 (14.6–26.7)	26.5 (17.8–37.6)	14.6 (12.8–16.7)	13.6 (11.5–16.0)
Any sick days (12 month), % (95%-CI)	69.3 (57.6–79.0)	74.5 (59.6–85.2)	**49.9 (47.0–52.8)**	**59.5 (56.0–62.9)**	68.1 (60.4–74.9)	76.5 (65.6–84.7)	**55.4 (52.5–58.1)**	**63.8 (60.7–66.8)**
No. of sick days if any^4^, mean (95%CI)	62.8 (38.0–87.6)	62.4 (24.9–100.0)	25.0 (20.5–29.5)	19.7 (16.3–23.0)	39.6 (27.4–51.8)	26.2 (16.6–35.9)	19.6 (17.4–21.7)	19.1 (15.1–23.1)

Bold type indicates significant differences between 1997–1999 and 2009–2012 (local significance level α = 0.05; see Table 5)

^1^German National Health Interview and Examination Survey 1998, mental health supplement (GHS-MHS, 1997–1999): weighted for population structure as of 12/31/1997; age range: 18–65

^2^German Health Interview and Examination Survey for Adults, mental health module (DEGS1-MH, 2009–2012): weighted for population structure as of 12/31/2010; age range: 18–65

^3^SF-36: Norm-based scoring

^4^In GHS-MHS *n* = 77 men and *n* = 166 women with MDD and *n* = 895 men and *n* = 1110 women without MDD reported any sick days in the last 12 months; in DEGS1-MH *n* = 49 men and *n* = 117 women with MDD and *n* = 828 men and *n* = 961 women without MDD reported any sick days in the last 12 months

The proportion of women with 12-month MDD reporting fair/poor self-rated health significantly decreased over time from 40.4% to 21.3% (odds ratio [OR] = 0.4, *p* = 0.001, see Table 5). This decrease was significantly more pronounced among women with 12-month MDD (*p* = 0.043) compared with the trend in women without MDD (OR = 0.7). However, the trends converged when effect estimates were adjusted for age (OR = 0.5 vs. OR = 0.7, *p* = 0.39, see Additional file 2). For men with MDD, there was no significant change in self-rated health over time (OR = 1.0) and no significant divergence (*p* = 0.37) from the trend in men without MDD (OR = 0.7).

**Table 5:** Effect estimates for time trends in health-related correlates: 1997–1999^1^ (reference) vs. 2009–2012^2^

		Men					Women				
		With MDD		Without MDD			With MDD		Without MDD		
		Effect estimate (95%-CI)	p _trend_	Effect estimate (95%-CI)	p _trend_	p _MDD × trend_	Effect estimate (95%CI)	p _trend_	Effect estimate (95%-CI)	p _trend_	p _MDD × trend_
Fair/poor self-rated health	OR	1.0 (0.5–2.1)	0.972	0.7 (0.5–0.9)	**0.006**	0.370	0.4 (0.2–0.7)	**0.001**	0.7 (0.6–0.9)	**0.007**	**0.043**
Health-related quality of life (4 weeks)											
Physical functioning	β	−1.5 (− 4.8–1.9)	0.391	0.7 (0.1–1.3)	**0.021**	0.214	1.5 (−1.1–4.1)	0.252	0.9 (0.4–1.5)	**0.001**	0.668
Physical role functioning	β	−4.3 (− 8.8–0.2)	0.061	−0.6 (−1.3–0.1)	0.098	0.116	0.2 (− 2.7–3.0)	0.897	−1.0 (− 1.6−−0.3)	**0.005**	0.433
Bodily pain	β	−1.7 (− 5.8–2.4)	0.413	3.0 (2.1–4.0)	**< 0.001**	**0.030**	6.1 (3.0–9.2)	**< 0.001**	3.3 (2.5–4.1)	**< 0.001**	0.077
General health	β	−0.6 (−4.1–2.9)	0.727	1.6 (0.9–2.3)	**< 0.001**	0.230	2.2 (−0.4–4.9)	0.097	1.4 (0.7–2.1)	**< 0.001**	0.534
Vitality	β	− 2.2 (− 6.4–1.9)	0.281	0.2 (−0.4–0.9)	0.448	0.237	0.7 (− 2.1–3.5)	0.612	0.4 (−0.2–1.1)	0.180	0.851
Social role functioning	β	−4.4 (− 8.9–0.0)	**0.048**	−0.2 (−0.8–0.4)	0.529	0.063	0.2 (−2.7–3.2)	0.870	−0.2 (− 0.9–0.6)	0.654	0.790
Emotional role functioning	β	−8.3 (−13.3−−3.3)	**0.001**	−1.3 (−1.9−−0.7)	**< 0.001**	**0.006**	−2.7 (− 6.7–1.2)	0.177	−2.6 (−3.4−−1.9)	**< 0.001**	0.966
Mental health	β	−2.0 (− 5.7–1.6)	0.273	0.5 (−0.1–1.2)	0.118	0.171	2.0 (−0.6–4.5)	0.131	1.0 (0.2–1.7)	**0.012**	0.482
*Physical component score*	β	−0.8 (−4.5–3.0)	0.694	1.6 (0.8–2.3)	**< 0.001**	0.248	3.8 (1.2–6.3)	**0.004**	1.8 (1.1–2.4)	**< 0.001**	0.113
*Mental component score*	β	−5.2 (−9.4−−1.1)	**0.014**	−0.7 (−1.3–0.0)	**0.040**	**0.033**	−1.2 (−4.6–2.2)	0.477	−0.9 (−1.8−−0.1)	**0.023**	0.874
Any days with activity limitation (4 weeks)											
due to mental health problems	OR	3.5 (1.4–9.0)	**0.010**	4.9 (2.0–11.7)	**< 0.001**	0.607	6.5 (2.8–15.4)	**< 0.001**	2.3 (1.3–4.2)	**0.007**	0.056
due to physical health problems	OR	1.4 (0.6–3.3)	0.482	0.9 (0.7–1.2)	0.403	0.363	1.5 (0.8–2.7)	0.251	0.9 (0.7–1.2)	0.494	0.196
Any sick days (12 month)	OR	1.3 (0.5–3.1)	0.569	1.5 (1.2–1.8)	**< 0.001**	0.787	1.5 (0.8–2.9)	0.198	1.4 (1.2–1.7)	**< 0.001**	0.846
No. of sick days if any	IRR	1.0 (0.5–2.1)	0.988	0.8 (0.6–1.0)	0.058	0.542	0.7 (0.4–1.1)	0.089	1.0 (0.8–1.2)	0.848	0.150

Models include MDD, time point and the interaction between MDD and time point (see Additional file 2 for age adjusted effect estimates). OR: Odds ratio from logistic regression (reference: 1997–1999); β: β coefficient from linear model; IRR: incidence rate ratio from negative binomial regression (reference: 1997–1999); p _trend_: p-value for testing a trend (test for OR/IRR = 1 or β = 0); p _MDD × trend_: p-value for testing differences in effect estimates of participants with MDD and without MDD (interaction). Bold type indicates significant results (local significance level α = 0.05)

^1^German National Health Interview and Examination Survey 1998, mental health supplement (GHS-MHS, 1997–1999): weighted for population structure as of 12/31/1997; age range: 18–65

^2^German Health Interview and Examination Survey for Adults, mental health module (DEGS1-MH, 2009–2012): weighted for population structure as of 12/31/2010; age range: 18–65

**Fig. 2:**
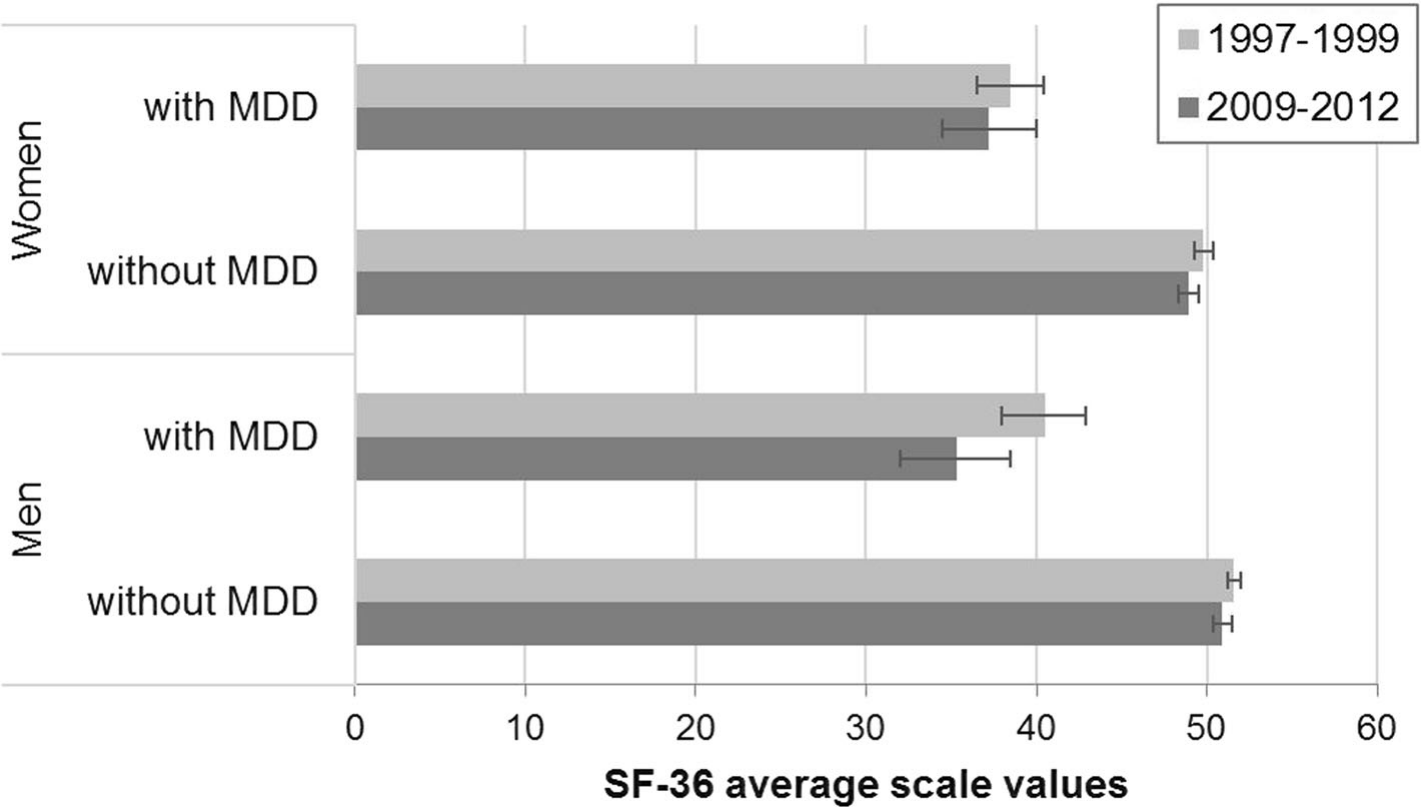
Health-related quality of life in 1997–1999 (GHS-MHS: German National Health Interview and Examination Survey 1998, mental health supplement, weighted for population structure as of 12/31/1997. Age range: 18–65) vs. 2009–2012 (DEGS1-MH: German Health Interview and Examination Survey for Adults, mental health module, weighted for population structure as of 12/31/2010. Age range: 18–65): Mental Component Score (based on SF-36 and norm-based scoring)

Health-related quality of life (past 4 weeks) changed over time: women with 12-month MDD showed a significant improvement for the physical component by an average of 3.8 points and a small but non-significant deterioration in the mental component (by −1.2 points), when comparing participants between 1997–1999 and 2009–2012. These trends did not significantly differ from female participants without MDD (*p* = 0.11 and *p* = 0.87, respectively). For men with 12-month MDD, there was no significant change over time in the physical component (by −0.8 points), but also no significant divergence (*p* = 0.25) from the improvement observed in men without MDD. In contrast, aggravation of the mental component over time in men with MDD (by −5.2 points) was significantly more pronounced (*p* = 0.033) compared with men without MDD (see Fig. 2). The outlined effect estimates for the total scales were comparable when adjusted for age, except for minor differences (see Additional file 2). The results for the total scales reflect changes regarding the SF-36 sub-scales. Women with 12-month MDD showed significant improvement in bodily pain over time (by 6.1 points). For all sub-scales, time trends did not significantly differ between women with and without MDD. In contrast, men showed a slight aggravation over time on all SF-36 subscales, reaching significance for social role functioning (by −4.4 points) and emotional role functioning (by −8.3 points). These time trends among men with MDD differed significantly from men without MDD in emotional role functioning (*p* = 0.006) and bodily pain (*p* = 0.030).

The risk of experiencing any days with activity limitation (past 4 weeks) due to mental health problems was significantly higher in 2009–2010 compared with 1997–1999 for men and women, whether MDD was present or not (with ORs ranging from 2.3 to 6.5). However, there was a trend towards a more pronounced increase in women with MDD compared with women without MDD (*p* = 0.056). In contrast, the risk of experiencing days with activity limitation due to physical health problems did not significantly change over time. The proportion of days with limitations due to physical health problems was already significantly higher in 1997–1999 compared with limitation days due to mental health problems. In 2009–2010, at least 25% of cases with 12-month MDD reported some days involving limitations due to each type of problem.

The risk of having any sick days (past 12 months) slightly rose over time (with ORs ranging from 1.3–1.5), but these changes were significant only for participants without MDD. In 2009–2010, approximately 75% of cases with 12-month MDD reported sick days. For women with 12-month MDD reporting any sick days, there was a trend towards a decreased number of sick days over time, from an average of 39.6 days to 26.2 days (incidence rate ratio 0.7, *p* = 0.089), but no change for women without MDD. For men with depression, there was no change over time in the number of sick days, but also no significant divergence (*p* = 0.54) from the decline in men without MDD (incidence ratio 0.8, *p* = 0.058, and incidence ratio 0.7, *p* = 0.005 when adjusted for age, see Additional file 2).

## Discussion

### Prevalence stability

Based on nationally representative samples of the general adult population, this study revealed no increase in overall 12-month MDD prevalence in Germany over a long period, irrespective of whether the data were standardized for demographic changes. Thus, the increased depression frequencies in health insurance data and increasing costs do not appear to reflect an “epidemic of depression” on a population level. Overall, the current results are in line with the initially presented meta-analysis and findings of the Global Burden of Disease Study, indicating no substantial change in MDD prevalence between 1990 and 2010, or 2005 and 2015, when adjusting for demographic changes [3, 7, 8]. However, women exhibited an MDD prevalence that was twice as high in the youngest age-group and a decrease in mild depression, corresponding to previous findings of increasing depression prevalence or chronicity in (younger) women [41–43].

Overall prevalence stability has previously been discussed as a potential outcome of improved prevention, mental health care and treatment benefits over time, possibly masking rising depression incidence or severity of illness [17, 44]. Meanwhile, exposure to risk factors for mental disorders in the population may have increased (e.g., higher levels of stress due to growing social inequality, isolation, urbanization and modernization in general; [2, 3]). International findings of increased lifetime depression prevalence and chronicity in younger cohorts appear to support this hypothesis [43, 45, 46]. However, societal changes could also result in reductions of depression risks (e.g., improved education). Furthermore, a general decrease in psychological distress or psychosocial stressors [47, 48], stable or even decreasing depression incidence [41, 43, 44], unchanged episode duration [44], and stable severity [49] have also been reported. A recent review concluded that there is no support for the first hypothesis of increasing exposure to risk factors in industrialized countries [17]. Likewise, potential changes in sociodemographic correlates were negligible for prevalence estimates in the current study, even within population subgroups (e.g., age groups). However, understanding the extent to which changes among women reflect growing exposure to risk factors over time within this specific subgroup requires further investigation.

### Rising mental health care need?

Changes in health-related correlates revealed increased disability over time in the MCS of SF-36, particularly among men with MDD, and an increased risk of experiencing days with activity limitation due to mental health problems. Thus, the current study provides some evidence of an increased need for mental health care for depression over time, particularly among men. Likewise, national health insurance data documents increasing work day loss attributable to depression (e.g. [50]), and treatment-seeking rates for major depression have been reported to be increasing internationally [51]. However, only relatively small changes were observed in the current study, and only slight improvement in non-help-seeking was reported for participants with mental disorders between 1997–1999 and 2009–2012 (62% vs. 57%) in Germany [52]. Thus, increasing depression frequency in health insurance data and growing costs cannot be explained solely by an increased need for mental health care in depression.

However, changes in health-related correlates also occurred in participants without MDD, while the perception of general health improved (see [37]). Likewise, international findings also indicate increasing mental health disability [53] and worsening of perceived mental health status [47, 48]. Thus, declining mental wellbeing may indicate an increasing need for mental health care in general (e.g., due to rising psychosocial demands over time). Another potential explanation is related to time trends in the process of reporting depressive symptoms themselves. The findings of several previous studies indicate that increased mental health literacy in the general population over time [54, 55] is associated with increased (public) awareness, recognition of psychological symptoms, and willingness to disclose [9, 11, 48, 56], as well as elevated help-seeking behavior [57, 58]. Thus, increased mental health literacy may have led to a more negative evaluation of stressors and perceived mental health over time, associated with rising subjective health care needs. International findings have indicated such a decrease in perceived mental health, while levels of distress were unchanged [47, 48, 53]. Having poor or fair mental health literacy has even been reported to be protective against MDD [9]. Furthermore, several results indicate age- and sex-dependent symptom expression [59–61], and mental health literacy [9]. Thus, even findings of increasing lifetime prevalence in younger cohorts or rising depression prevalence among younger women may be specifically associated with increased reporting of symptoms. However, only minor changes in health-related correlates were observed overall, and little evidence of rising mental health literacy is available in view of the enormous increases in national health insurance data (see [17]).

### Limitations

The following potential limitations should be considered interpreting the findings of the current study. Underestimation of MDD prevalence at both time points may have been caused by “recall bias”, selective non-response of less healthy participants and exclusion of institutionalized individuals in both surveys [21, 22, 24]. Thus, people with severe depression may have been particularly underrepresented. In addition, participants’ “reporting bias” and varying diagnostic accuracy between population subgroups may have led to prevalence underestimation in older participants and male participants [62, 63]. Moreover, the inclusion of some longitudinal data could lead to an underestimation of MDD prevalence in 2009–2012, due to a potentially higher rate of re-participation among healthier participants. Moreover, in the statistical analysis the two survey populations were considered to be independent, neglecting potential correlations related to re-participation of some participants in the DEGS1-MH. The particularly small number of men with MDD in the surveys resulted in low statistical power for detecting time trends within this subgroup. A further potential limitation could be related to the time lags between the core surveys and mental health supplements, which may have led to an underestimation of associations between 12-month MDD and health-related correlates. However, associations may also have been overestimated due to a construct overlap of depressive symptoms (e.g., energy loss) with outcome measures such as SF-36 and self-reported disability.

## Conclusions

The current study provides valid, up-to-date information about time trends in depression prevalence, severity and symptoms in the general population in Germany over a long period with a high-quality diagnostic level. To date, national cross-sectional data has been lacking comparability over time due to divergent measures or diagnostic algorithms. Moreover, this is the first study comparing DSM-IV-based major depression prevalence over time while also considering health-related correlates. Furthermore, the current study also provides an evaluation of time trends in view of demographic changes in the underlying population. Thus, the current findings contribute significantly to the ongoing national and international debate regarding the potential increase of depression in western countries.

In contrast to the frequently claimed “epidemic” of depression, we found stable overall prevalence in Germany between 1997–1999 and 2009–2012. Demographic changes had a marginal impact on the examined time trends within the considered age range. In conclusion, increased depression frequencies in national health insurance data and associated growing health care costs are not attributable to overall prevalence changes at a population level.

However, shifted age distribution and increased severity among women may reflect a rising depression risk within this specific subgroup. Furthermore, we found some evidence for an increased need for mental health care for depression over time, particularly among men. However, changes in mental wellbeing also occurred in the general population, which may have also contributed to an increase in depression diagnoses in the health care system. Thus, the observed time trends suggest the need for further investigations of potentially rising psychosocial demands in the general population, and specifically increasing depression risks among women, considering age- and sex-specific developments in mental health literacy.

Finally, divergent time trends in primary and secondary data indicate the need for a critical review of mental health care in Germany, rising questions about the effects of simultaneously expanded services and provision of treatment (e.g. [64]). Jorm AF, Patten SB, Brugha TS and Mojtabai R [17] already highlighted the impact of a “treatment gap”, “quality gap” and “prevention gap” for the lack of improvement in population prevalence. In Germany, only 34.6% of participants with 12-month MDD reported any service use due to mental health problems in 2009–2012 [65]. Locally varying access to mental health care has previously been identified as an important determinant of help-seeking behavior [66, 67]. In addition, increasing acceptance of mental health care services [55] and rising public knowledge appear to not have resulted in improved social acceptance of people with mental illness over time [54]. Furthermore, targeting of treatment seems to be questionable: concordance of self-reported clinician diagnosed depression and DSM-IV-based MDD diagnosis is remarkably low [33, 62]. False positive depression diagnoses were particularly high in primary care [68], also indicating “over-representation” of depression within the German health care system, concurrent with the persisting treatment gap. Moreover, national health insurance data provides evidence for lacking quality of treatment [69–73]. These findings suggest that public health initiatives in Germany should continue to reduce access barriers to mental health care services, and focus on improving targeting and quality of treatment for depression. Furthermore, primary and secondary data seem to fundamentally lack comparability with regard to their respective depression indicators. Thus, the current findings emphasize the potential benefits of linking secondary data regarding health care utilization and service provision with standardized measures of depression based on primary data.

## Additional files

Additional file 1: Prevalence of depression symptoms in cases with 12-month MDD 1997–1999 vs. 2009–2012 (PDF 127 kb)

Additional file 2: Age-adjusted effect estimates for time trends in health-related correlates: 1997–1999 (reference) vs. 2009–2012 (PDF 95 kb)

## Abbreviations

CIDI: Composite International Diagnostic Interview
DEGS1: German Health Interview and Examination Survey for Adults
DEGS1-MH: Mental health module of DEGS1
DSM-IV: Diagnostic and Statistical Manual of Mental Disorders, 4th edition
DSM-IV-TR: Diagnostic and Statistical Manual of Mental Disorders, 4th edition, Text Revision
GHS: German National Health Interview and Examination Survey 1998
GHS-MHS: Mental health supplement of GHS
MCS: Mental component score
MDD: Major depressive disorder
PCS: Physical component score
SES: Socio-economic status
SF-36: Short Form 36
