# Supplementary material for: Time trends in depression prevalence and health-related correlates: results from population-based surveys in Germany 1997–1999 vs. 2009–2012
Source: BMC Psychiatry. 2018 Dec 20;18:394. doi: 10.1186/s12888-018-1973-7 (PMC6302526; doi:10.1186/s12888-018-1973-7)
Supplement: Supplementary file 1 — Prevalence of depression symptoms in cases with 12-month MDD 1997–1999 vs. 2009–2012 (PDF 127 kb) [file 12888_2018_1973_MOESM1_ESM.pdf]

Additional file 1 Prevalence of depression symptoms in cases with 12-month MDD 1997–1999[1] vs. 2009–2012[2]

	**Men**			**Women**		
	**1997–1999 (n = 110)**	**2009–2012 (n = 71)**	**p-value[3]**	**1997–1999 (n = 238)**	**2009–2012 (n = 159)**	**p-value[3]**
**Depression symptoms**, % (95% CI)						
Depressed mood	89.2 (81.1–94.0)	92.0 (79.8–97.1)	0.602	92.6 (87.8–95.6)	93.9 (89.0–96.7)	0.627
Diminished interest/pleasure	71.6 (59.6–81.1)	68.3 (54.2–79.7)	0.691	**78.2 (70.5–84.4)**	**63.7 (52.7–73.3)**	**0.018**
Significant weight/appetite change	49.1 (38.4–59.8)	60.5 (46.7–72.8)	0.209	**60.3 (52.2–67.9)**	**82.0 (74.4–87.8)**	**<0.001**
Insomnia/hypersomnia	97.5 (93.0–99.2)	97.3 (89.7–99.3)	0.924	90.6 (84.2–94.6)	96.1 (91.2–98.3)	0.052
Psychomotor agitation/retardation	45.1 (34.3–56.3)	41.5 (29.0–55.2)	0.694	49.3 (41.8–56.9)	52.3 (40.7–63.7)	0.650
Fatigue/loss of energy	83.6 (72.7–90.7)	90.8 (80.8–95.8)	0.236	92.5 (88.0–95.5)	91.2 (84.0–95.3)	0.696
Guilt/worthlessness	**53.1 (42.6–63.4)**	**71.4 (57.2–82.4)**	**0.035**	**63.9 (56.9–70.4)**	**79.4 (68.6–87.2)**	**0.019**
Diminished ability to concentrate	94.2 (87.8–97.3)	88.9 (77.5–94.9)	0.213	84.4 (77.0–89.7)	86.8 (78.4–92.3)	0.612
Suicidality	53.5 (41.5–65.1)	58.0 (44.3–70.5)	0.619	70.9 (64.8–76.4)	64.4 (53.7–73.8)	0.258

[1] German National Health Interview and Examination Survey 1998, mental health supplement (GHS-MHS, 1997–1999): weighted for population structure as of 12/31/1997; age range: 18–65.
[2] German Health Interview and Examination Survey for Adults, mental health module (DEGS1-MH, 2009–2012): weighted for population structure as of 12/31/2010; age range: 18–65.
[3] p-value based on Rao-Scott chi-square test. Bold type indicates significant differences between 1997–1999 and 2009–2012 (local significance level $\alpha = 0.05$).
