# Supplementary material for: Time trends in depression prevalence and health-related correlates: results from population-based surveys in Germany 1997–1999 vs. 2009–2012
Source: BMC Psychiatry. 2018 Dec 20;18:394. doi: 10.1186/s12888-018-1973-7 (PMC6302526; doi:10.1186/s12888-018-1973-7)
Supplement: Supplementary file 2 — Age-adjusted effect estimates for time trends in health-related correlates: 1997–1999 (reference) vs. 2009–2012 (PDF 95 kb) [file 12888_2018_1973_MOESM2_ESM.pdf]

**Additional file 2** Age-adjusted effect estimates for time trends in health-related correlates: 1997–1999[1] (reference) vs. 2009–2012[2]

| | | Men | | | | | Women | | | | |
|---|---|---|---|---|---|---|---|---|---|---|---|
| | | With MDD | | Without MDD | | | With MDD | | Without MDD | | |
| | | **Effect estimate (95% CI)** | **$p_{trend}$** | **Effect estimate (95% CI)** | **$p_{trend}$** | **$p_{MDD \times trend}$** | **Effect estimate (95% CI)** | **$p_{trend}$** | **Effect estimate (95% CI)** | **$p_{trend}$** | **$p_{MDD \times trend}$** |
| **Fair/poor self-rated health** | OR | 0.9 (0.4–1.8) | 0.738 | 0.6 (0.5–0.8) | **<0.001** | 0.384 | 0.5 (0.3–0.9) | **0.022** | 0.7 (0.5–0.9) | **0.002** | 0.386 |
| **Health-related quality of life (4 weeks)** | | | | | | | | | | | |
| Physical functioning | β | −1.0 (−4.0–2.0) | 0.530 | 1.0 (0.5–1.6) | **<0.001** | 0.200 | 0.3 (−2.1–2.8) | 0.790 | 1.2 (0.6–1.7) | **<0.001** | 0.500 |
| Physical role functioning | β | −3.9 (−8.0–0.3) | 0.069 | −0.3 (−0.9–0.4) | 0.378 | 0.103 | −0.7 (−3.5–2.1) | 0.622 | −0.8 (−1.4–−0.1) | **0.032** | 0.970 |
| Bodily pain | β | −1.3 (−5.2–2.6) | 0.513 | 3.4 (2.4–4.3) | **<0.001** | **0.026** | 5.1 (2.2–8.1) | **0.001** | 3.5 (2.7–4.4) | **<0.001** | 0.301 |
| General health | β | −0.3 (−3.5–2.9) | 0.872 | 1.9 (1.2–2.5) | **<0.001** | 0.213 | 1.5 (−1.1–4.0) | 0.261 | 1.6 (0.9–2.2) | **<0.001** | 0.931 |
| Vitality | β | −2.2 (−6.4–1.9) | 0.284 | 0.2 (−0.4–0.9) | 0.462 | 0.240 | 0.9 (−1.9–3.7) | 0.543 | 0.4 (−0.2–1.1) | 0.204 | 0.763 |
| Social role functioning | β | −4.4 (−8.7–0.0) | **0.050** | −0.1 (−0.8–0.5) | 0.679 | 0.061 | 0.1 (−2.9–3.1) | 0.947 | −0.1 (−0.9–0.6) | 0.720 | 0.880 |
| Emotional role functioning | β | −8.1 (−13.0–−3.3) | **0.001** | −1.1 (−1.8–−0.5) | **<0.001** | **0.005** | −2.9 (−6.9–1.0) | 0.143 | −2.6 (−3.3–−1.8) | **<0.001** | 0.858 |
| Mental health | β | −2.0 (−5.6–1.6) | 0.276 | 0.6 (−0.1–1.2) | 0.116 | 0.171 | 1.9 (−0.6–4.5) | 0.128 | 1.0 (0.2–1.8) | **0.013** | 0.481 |
| *Physical component score* | β | −0.2 (−3.7–3.2) | 0.887 | 1.9 (1.3–2.6) | **<0.001** | 0.230 | 2.5 (0.1–4.9) | **0.045** | 2.0 (1.4–2.7) | **<0.001** | 0.711 |
| *Mental component score* | β | −5.3 (−9.5–−1.1) | **0.014** | −0.8 (−1.4–−0.1) | **0.024** | **0.035** | −0.9 (−4.3–2.5) | 0.620 | −1.0 (−1.8–−0.2) | **0.014** | 0.920 |
| **Any days with activity limitation (4 weeks)** | | | | | | | | | | | |
| due to mental health problems | OR | 3.6 (1.4–9.3) | **0.010** | 5.0 (2.0–12.2) | **0.001** | 0.609 | 6.1 (2.6–14.3) | **<0.001** | 2.3 (1.3–4.3) | **0.006** | 0.072 |
| due to physical health problems | OR | 1.4 (0.6–3.4) | 0.469 | 0.9 (0.7–1.2) | 0.458 | 0.368 | 1.4 (0.7–2.6) | 0.297 | 0.9 (0.7–1.2) | 0.534 | 0.239 |
| **Any sick days (12 month)** | OR | 1.4 (0.6–3.3) | 0.498 | 1.5 (1.3–1.9) | **<0.001** | 0.794 | 1.3 (0.7–2.5) | 0.439 | 1.5 (1.2–1.8) | **<0.001** | 0.688 |
| **No. of sick days if any** | IRR | 1.0 (0.6–1.9) | 0.907 | 0.7 (0.6–0.9) | **0.005** | 0.294 | 0.7 (0.4–1.1) | 0.090 | 0.9 (0.7–1.1) | 0.379 | 0.241 |

Models include MDD, time point, age, and the interaction between MDD and time point. OR: Odds ratio from logistic regression (reference: 1997–1999); β: β coefficient from linear model; IRR: incidence rate ratio from negative binomial regression (reference: 1997–1999); $p_{trend}$: p-value for testing a trend (test for OR/IRR = 1 or β = 0); $p_{MDD \times trend}$: p-value for testing differences in effect estimates of participants with MDD and without MDD (interaction). Bold type indicates significant results (local significance level α = 0.05).

[1] German National Health Interview and Examination Survey 1998, mental health supplement (GHS-MHS, 1997–1999): weighted for population structure as of 12/31/1997; age range: 18–65.

[2] German Health Interview and Examination Survey for Adults, mental health module (DEGS1-MH, 2009–2012): weighted for population structure as of 12/31/2010; age range: 18–65.
